# Ligand entry in human ileal bile acid-binding protein is mediated by histidine protonation

**DOI:** 10.1038/s41598-019-41180-7

**Published:** 2019-03-18

**Authors:** Gergő Horváth, Orsolya Egyed, Changguo Tang, Mihály Kovács, András Micsonai, József Kardos, Orsolya Toke

**Affiliations:** 1 Laboratory for NMR Spectroscopy, Research Centre for Natural Sciences, Hungarian Academy of Sciences, 2 Magyar tudósok körútja, H-1117 Budapest, Hungary; 2 Department of Biochemistry and Molecular Biophysics, Washington University School of Medicine, 660 South Euclid Avenue, St. Louis, Missouri, 63110 USA; 3 Department of Biochemistry, ELTE-MTA “Momentum” Motor Enzymology Research Group, Eötvös Loránd University, Pázmány Péter sétány 1/C, H-1117 Budapest, Hungary; 4 Department of Biochemistry, MTA-ELTE NAP B Neuroimmunology Research Group, Institute of Biology, Eötvös Loránd University, Pázmány Péter sétány 1/C, H-1117 Budapest, Hungary

## Abstract

Human ileal bile acid-binding protein (hI-BABP) has a key role in the intracellular transport of bile salts. To explore the role of histidine protonation in the binding process, the pH-dependence of bile salt binding and internal dynamics in hI-BABP was investigated using NMR spectroscopy and biophysical tools. Thermodynamic and kinetic measurements show an increase in the overall binding affinity and the association rate constant of the first binding step below the pK_a_ of the histidines, suggesting that ligand binding is favoured by the protonated state. The overlap between residues exhibiting a high sensitivity to pH in their backbone amide chemical shifts and protein regions undergoing a global ms conformational exchange indicate a connection between the two processes. According to ^15^N NMR relaxation dispersion analysis, the slow motion is most pronounced at and above the pK_a_ of the histidines. In agreement with the NMR measurements, MD simulations show a stabilization of the protein by histidine protonation. Hydrogen-bonding and van der Waals interactions mediating the flow of information between the C/D- and G/H-turn regions hosting the three histidines, suggest a complex way of pH-governed allosteric regulation of ligand entry involving a transition between a closed and a more open protein state.

## Introduction

Intracellular lipid binding proteins (iLBPs) are small, 14–15 kDa polypeptide chains that are thought to function in the transcellular trafficking of fatty acids, retinoids, and bile salts^1,2^. Additionally, there is increasing evidence that they have a role in stimulating the transcriptional activities of nuclear receptors initiated by a ligand-induced nuclear translocation^3–5^. The molecular mechanism by which iLBPs regulate the transport of lipid-like compounds within the cell is not yet fully understood. One of the fundamental questions is the way of ligand entry and exit and how ligand transfer is accomplished between the protein and the cell membrane.

**Figure 1:**
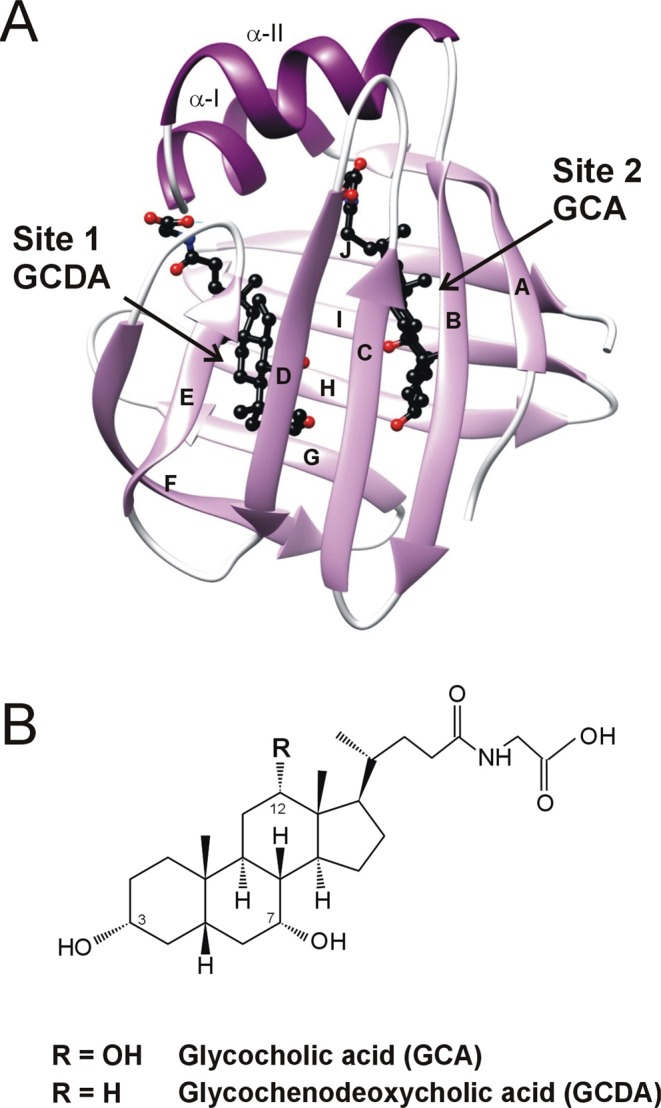
(**A**) Ribbon diagram of the heterotypic human I-BABP:GCDA:GCA complex determined by solution NMR (PDB entry 2MM3^6^). Bound bile salts are shown in a ball-and-stick representation. (**B**) Chemical structure of glycocholic acid (GCA) and glycochenodeoxycholic acid (GCDA), the two most abundant bile salts in humans.

Despite the variability of their lipophilic ligands and the divergence in their amino acid sequence, iLBPs share a common topology comprised of two orthogonal antiparallel beta-sheets and a helix-loop-helix motif covering the beta-barrel (Fig. 1A)^1,6^. The binding cavity of ~1000Å^3^ is located inside of the beta-barrel. Internal dynamics appear to have a major role in iLBP-ligand interactions^7^. Stopped-flow kinetic studies of both fatty acid binding proteins (FABP)^8^ and bile acid binding proteins (BABP)^9^ indicate a rate-limiting conformational change preceding the binding step. Moreover, NMR relaxation measurements show evidence of the presence of µs-ms conformational fluctuations with a possible role in mediating ligand entry in FABPs^10^, cellular retinol binding proteins (CRBPs)^11^, and BABPs^12,13^. Despite the common topology, there are substantial differences between the three subfamilies regarding the protein regions involved in the fluctuations. In FABPs, the helical segments together with the proximal C/D-turn are thought to be part of a dynamic portal region mediating access to the enclosed binding cavity^10^. Similarly, in some of the reported CRBP structures, the helical region exhibiting considerable disorder in the *apo* state has been found to undergo a millisecond timescale fluctuation in the absence of ligand^11,14^. More recent MD simulation studies of CRBP 1 and 2 indicate two different entry sites for the ligand, both involving the helical cap and proximal loop regions^15^. In unligated BABPs, both in the human and the chicken analogues, the E/F and the G/H regions of the C-terminal half exhibit the most intense slow internal motion and are hypothesized to form an alternate portal region^13,16^. Additionally, in human ileal bile acid-binding protein (hI-BABP), there is a second cluster of residues involving the C/D-turn and the B and D beta-strands undergoing an exchange process with a slightly different exchange rate^17^, the functional relevance of which is yet unclear. Temperature-dependent relaxation dispersion (R_ex_) measurements show a strong enthalpy-entropy compensation for both clusters between the ground and a sparsely populated higher energy state characteristic of order-disorder transitions^17^. More recently, a joint analysis of R_ex_ measurements and NMR thermal melting data has indicated a structural and thermodynamic connection between ms timescale conformational fluctuations at and below room temperature (10–25 °C) and thermal unfolding (60 °C)^18^. Supporting the NMR relaxation data, MD simulations show evidence of correlated motions involving the helical cap, the E-F strands, and the bottom of the β-barrel. Additionally, the calculations capture a partially unfolded state with an open EF-region and an increased hydrophobic surface, posing an aggregation risk for the protein in the absence of ligands^18^.

Besides mediating ligand entry, conformational flexibility and slow internal fluctuations have also been suggested to have a role in transmitting information between the two binding sites of human I-BABP, a protein displaying a moderate-to-high degree of positive binding cooperativity in its interactions with di- and trihydroxy bile salts (Fig. 1B)^19–21^. Specifically, stopped-flow kinetic studies of human I-BABP indicate that positive cooperativity is related to a slow conformational change of the protein (~30–50 s^−1^ at 25 °C) occurring after the second binding step^9^.

Proton transfer mechanisms and tautomer equilibria near physiological pH may have a role in conformational transitions associated with ligand entry and/or a subsequent communication between the binding sites, in particular that hydrogen bond networks have been shown to be key determinants of positive binding cooperativity in human I-BABP^21^. Due to their multiple tautomeric and charged states, histidines play an important role in binding processes in proteins, in particular that the pK_a_ of their imidazole ring is usually close to physiological pH allowing them to act as either acids or bases^22,23^. In human I-BABP, there are three histidine residues, two in the C/D-turn (H52, H57) and one in the H beta strand (H98) that is within regions undergoing slow conformational fluctuations in the *apo* state. Both in the human ileal and the chicken liver analogues, slow motions cease upon bile salt binding suggesting that ligation stabilizes one of the two conformations^12,13,17^. Moreover, in chicken liver bile acid-binding protein (cL-BABP), a buried histidine in the G/H-region has been indicated in an opening/closing motion^12^ and a possible role in the cooperativity between the two binding sites as well^24^. In human I-BABP, with additional histidines in the C/D-turn region, the exchange dynamics appears to be more complex with two subsets of residues exhibiting different exchange kinetics^17^. To explore the role of histidine protonation in human I-BABP-bile salt recognition and gain more insight into the functional role of conformational fluctuations in the protein, we performed a pH-dependent study of the thermodynamics and kinetics of bile salt binding in conjunction with NMR relaxation measurements and MD simulations. Our results unveil an intimate relation between histidine protonation and an opening/closing motion mediating ligand entry in the *apo* form of hI-BABP raising the possibility of a pH-dependent mechanism pf bile salt uptake in the enterocytes of the distal small intestine.

## Results

### Titration calorimetry experiments to characterize the thermodynamics of bile salt binding

**Figure 2:**
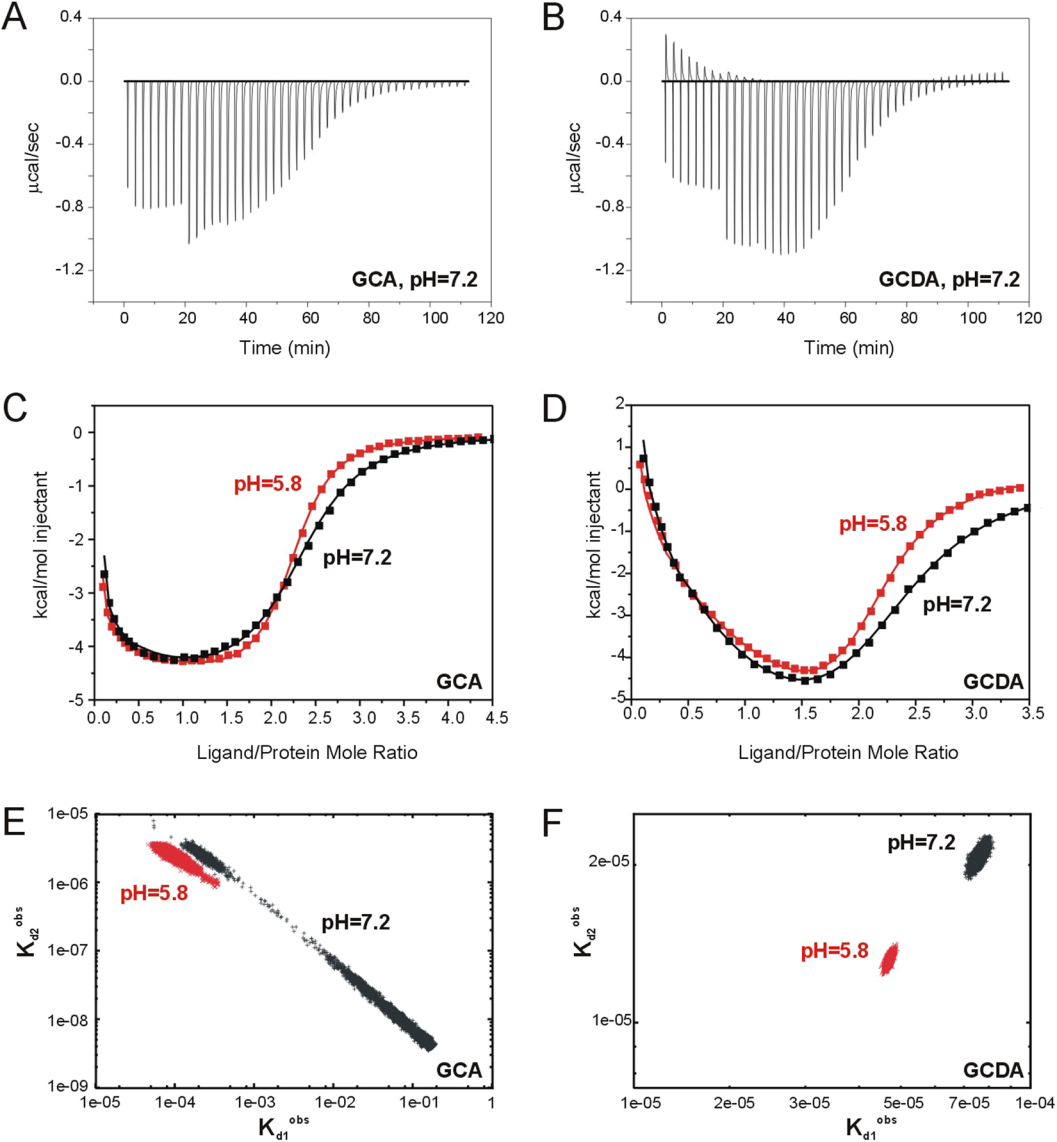
ITC analysis of the pH-dependence of bile salt binding to human I-BABP. Injection profiles for (**A**) GCA and (**B**) GCDA at pH = 7.2. The discontinuity at an x axis value of approximately 17 min represents a change in the injection volume from 0.4 to 0.8 µL. Least-squares fitted binding isotherms for (**C**) GCA and (**D**) GCDA at pH = 5.8 (red) and 7.2 (black). The curve through the points of the isotherms corresponds to a least-squares fit of the raw data using the stepwise binding model defined in eq. 1 with parameters listed in Table 1. Scatter plot of K_d2_^obs^ versus K_d1_^obs^ for the binding of (**E**) GCA and (**F**) GCDA to human I-BABP at the two investigated values of pH as obtained from the Bayesian analysis of the probability distribution for the fitted stepwise binding parameters. A Markov chain Monte Carlo method was used to sample 10000 points from the posterior probability distribution of K_d1_^obs^, K_d2_^obs^, ΔH_1_^obs^, and ΔH_2_^obs^ given the calorimetry data. The last 9000 points plotted here for each dataset were used to obtain the average values of the parameters listed in Table 1.

Injection profiles and binding isotherms for the interactions of human I-BABP with glycocholic acid (GCA) and glycochenodeoxycholic acid (GCDA), the two most abundant bile salts in humans, at pH = 5.8 and pH = 7.2 are shown in Fig. 2. Similar to previous findings, a biphasic binding profile was obtained for both interactions. To quantitate the thermodynamic parameters of ligand binding, isothermal titration calorimetry (ITC) data sets were fit to the stepwise binding model shown in eq. 1 by Bayesian analysis. Initial values for the parameter search were obtained from nonlinear least-squares fitting. As it was described earlier^19^, Bayesian analysis overcomes the problem of underestimating the parameter space consistent with the data, a commonly encountered difficulty in nonlinear least-squares analysis of systems with positive cooperativity. The dissociation constants and stepwise binding enthalpies together with the calculated Hill coefficients of positive cooperativity, are listed in Table 1. Bayesian analysis of the probability distribution for the fitted stepwise dissociation constants for the binding of GCA and GCDA at the two different values of pH is shown in Fig. 2E,F. The obtained stepwise binding parameters at pH = 7.2 are in reasonable agreement with previously reported values^20,21^ taken into consideration the difficulty of the determination of ΔH_1_ in highly cooperative systems even with Bayesian analysis^19^.

**Table 1:** Stepwise binding parameters for the interaction of human I-BABP with GCA and GCDA at pH = 5.8 and pH = 7.2 (20 mM potassium phosphate, 135 mM KCl, 10 mM NaCl, 0.05% NaN_3_, 25 °C).

	n_H_^a^	K_d1_°^bs^ (μM)	K_d2_°^bs^ (μM)	ΔH°_1_°^bs^ (kcal/mol)	ΔH^o^_2_^obs^ (kcal/mol)	TΔS_1_^obs^ (kcal/mol)	TΔS_2_^obs^ (kcal/mol)
GCA							
pH = 5.8	1.74 ± 0.01	105 ± 30	2.4 ± 0.4	−1.3 ± 0.7	−9.3 ± 0.7	4.1 ± 0.2	−1.6 ± 0.1
pH = 7.2	1.78 ± 0.01	276 ± 80	4.3 ± 0.9	−0.3 ± 0.9	−10.0 ± 0.1	4.6 ± 0.2	−2.7 ± 0.1
GCDA							
pH = 5.8	1.31 ± 0.01	47 ± 6	13.2 ± 0.3	0.86 ± 0.03	−9.9 ± 0.1	6.76 ± 0.08 -	−3.24 ± 0.01
pH = 7.2	1.31 ± 0.01	76 ± 2	21 ± 1	2.0 ± 0.1	−12.1 ± 0.1	7.62 ± 0.02	−5.72 ± 0.03

^a^The Hill coefficient is related to the stepwise binding parameters as follows: n_H_ = 2/[1 + (K_d2_^obs^/K_d1_^obs^)^1/2^].

As it is revealed by the products of K_d1_ and K_d2_, a slight increase in the overall binding affinity is observed for both bile salts by lowering the pH, which arises from both enthalpic (ΔH_1_ = 0.86 ± 0.03 kcal/mol at pH = 5.8 *vs*. ΔH_1_ = 2.0 ± 0.1 kcal/mol at pH = 7.2 for GCDA and ΔH_1_ = −1.3 ± 0.7 kcal/mol at pH = 5.8 *vs*. ΔH_1_ = −0.3 ± 0.9 kcal/mol at pH = 7.2 for GCA) and entropic (TΔS_2_ = −3.24 ± 0.01 kcal/mol at pH = 5.8 *vs*. TΔS_2_ = −5.72 ± 0.03 kcal/mol at pH = 7.2 for GCDA and TΔS_2_ = −1.6 ± 0.1 kcal/mol at pH = 5.8 *vs*. TΔS_2_ = −2.7 ± 0.1 kcal/mol at pH = 7.2 for GCA) effects. The large negative enthalpic contribution of the second binding step reflecting bile salt-amino acid interactions as well as, most likely, interactions of bile salts with bound water molecules within the binding cavity, shows a slightly more pH sensitivity for the dihydroxy bile salt (ΔH_2_ = −9.9 ± 0.1 kcal/mol at pH = 5.8 *vs*. ΔH_2_ = −12.1 ± 0.1 kcal/mol at pH = 7.2) than for GCA (ΔH_2_ = −9.3 ± 0.7 kcal/mol at pH = 5.8 *vs*. ΔH_2_ = −10.0 ± 0.1 kcal/mol at pH = 7.2).

Regarding positive cooperativity, the difference between the Hill coefficients at pH = 5.8 and 7.2 observed for GCA (trihydroxy bile salt with more pronounced positive binding cooperativity) appears to be within experimental error, whereas in the case of GCDA, there is no detectable change at all in binding cooperativity in the investigated pH range. This is likely a manifestation of a strong entropy-enthalpy compensation in the system, which appears to be more pronounced for the binding of the dihydroxy bile salt.

### Ligand binding kinetics

**Figure 3:**
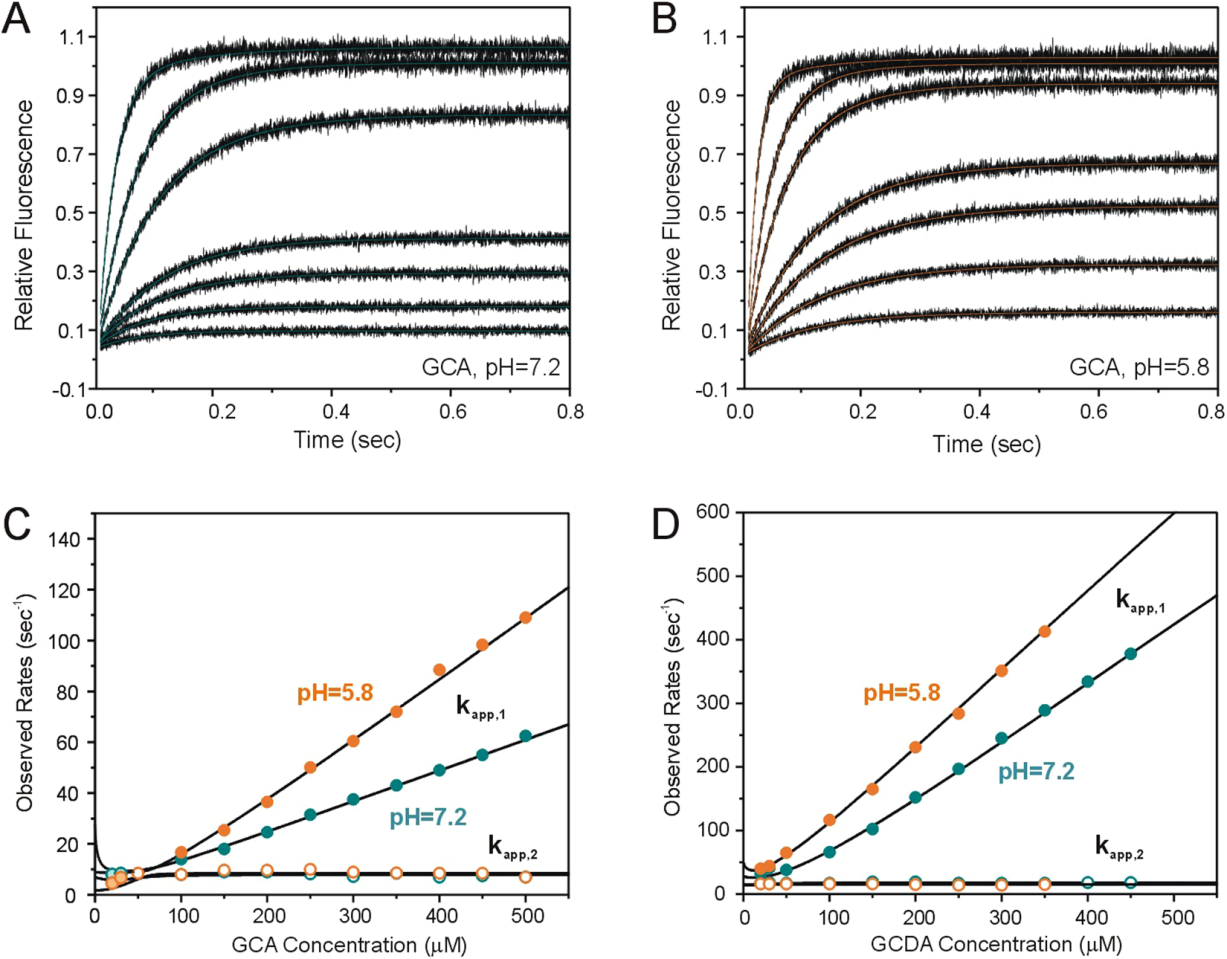
Stopped-flow kinetic analysis of the pH-dependence of bile salt binding to human I-BABP. (**A**,**B**) Representative stopped-flow traces showing fluorescence changes upon mixing human I-BABP with GCA in buffer containing 20 mM potassium phosphate, 135 mM KCl, 10 mM NaCl, and 0.05% NaN_3_ at (**A**) pH = 7.2 and (**B**) pH = 5.8 at 15 °C (λ_ex_ = 290 nm, λ_em_ > 320 nm). Final protein concentration was 1 µM. Final bile salt concentrations were 20, 30, 40, 50, 100, 150, 300 µM. The curve through the data points corresponds to a least-squares fit using a double exponential with observed rates depicted in (**C**). (**C**,**D**) Observed rates for the binding of (**C**) GCA and (**D**) GCDA to human I-BABP as a function of ligand concentration as determined from the stopped-flow experiments performed at pH = 5.8 (orange) and 7.2 (green). The solid lines are nonlinear least-squares fits according to the four-step sequential binding mechanism defined in eq. 3 with rate constants listed in Table 2.

Stopped-flow fluorescence experiments were used to reveal the effect of pH on the kinetics of human I-BABP-bile salt interaction. The experiments have been performed under pseudo-first-order conditions by mixing the protein with an excess of ligand. Stopped-flow traces showing fluorescence changes upon GCA and GCDA binding to human I-BABP are shown in Fig. 3. As it has been shown previously^9^, the kinetic mechanism of bile salt binding can be described in a four-step sequential model according to eq. 3 (Materials and Methods). Accordingly, a rate-limiting conformational transition is followed by two sequential binding steps, and an additional slow conformational change. Stopped-flow traces shown in Fig. 3A,B can most adequately be fitted by a biexponential (eq. 2), with the exponents corresponding to the two observed rates (eigenvalues). Among the two, *k*_*app,1*_ can be given in a polynomial function of the ligand concentration, which at high values of [L] becomes nearly linear. Under these conditions, the rate constant of the association (k_2_ in eq. 3) can be estimated from the slope of the curve. The other observed rate, *k*_*app,2*_ (k_−4_ in eq. 3) shows no change (within experimental error) with bile salt concentration and as it has been shown earlier corresponds to a slow conformational change following the second binding step associated with positive binding cooperativity.

The observed rates obtained from the biexponential fits for GCA and GCDA at pH = 5.8 and 7.2 (15 °C) are shown in Fig. 3C,D. Shifting the pH toward more acidic conditions markedly increases the association rate for both bile salts, whereas the rate of the unimolecular step remains unchanged. The solid lines are least-square fits of the observed rates according to the mechanism shown in eq. 3 with parameters listed in Table 2. Observed rates were calculated as eigenvalues of coefficient matrices as a function of ligand concentration using Mathematica. Initial values for the fits were obtained from our previous analysis at 25 °C and pH = 7.2^9^. Similarly to our previous findings at room temperature, the association rate constant of the first binding step (k_2_) is substantially faster for GCDA than for GCA and the difference between the two bile salts is maintained at both values of pH. For GCA, the increased value of k_2_ at more acidic conditions parallels with a faster dissociation rate constant (k_−2_) yielding a similar ratio of k_−2_/k_2_ at the two investigated pHs at this temperature. For GCDA, k_−2_ is significantly smaller at lower pH. Regarding the second binding step, the decrease in pH appears to have a significantly larger effect on the binding kinetics of the trihydroxy bile salt with slower association (k_3_) and dissociation rates (k_−3_) under more acidic conditions. Finally, the interconversion between PL_2_ and PL_2_^*^ shows nearly the same kinetics at pH = 7.2 and pH = 5.8 for both bile salts. Similar to our previous observation at room temperature, incorporation of an initial unimolecular step (P’ ↔ P) slightly improved the quality of the fits but had no effect on the value of the rate constants for the subsequent steps. We note that the time scale of the P’ ↔ P transition matches well the value of k_ex_ inferred from relaxation dispersion NMR measurements in the *apo* form^17^ between pH = 6.3–8.0 (*cf below*).

**Table 2:** Kinetic parameters characterizing the binding of GCA and GCDA to human I-BABP according to eq. 3 at pH = 5.8 and 7.2 (20 mM potassium phosphate, 135 mM KCl, 10 mM NaCl, 0.05% NaN_3_, 15 °C).

	GCA		GCDA	
	pH = 5.8	pH = 7.2	pH = 5.8	pH = 7.2
k_1_ (s^−1^)	1900 ± 400	1400 ± 300	1900 ± 400	1400 ± 300
k_−1_ (s^−1^)	200 ± 70	170 ± 80	200 ± 70	170 ± 80
k_2_ (s^−1^ μM^−1^)	0.28 ± 0.03	0.14 ± 0.03	1.48 ± 0.04	1.22 ± 0.02
k_−2_ (s^−1^)	64 ± 7	28 ± 5	73 ± 22	164 ± 29
k_3_ (s^−1^ μM^−1^)	1.1 ± 0.2	6.4 ± 0.3	2.8 ± 0.1	2.2 ± 0.2
k_−3_ (s^−1^)	2.2 ± 0.5	38 ± 4	50 ± 3	24 ± 3
k_4_ (s^−1^)	1.1 ± 0.2	0.8 ± 0.1	0.2 ± 0.1	1.4 ± 0.2
k_−4_ (s^−1^)	7.4 ± 0.6	7.0 ± 0.8	14.9 ± 0.8	16.5 ± 0.9

### pH response of backbone amide chemical shifts

**Figure 4:**
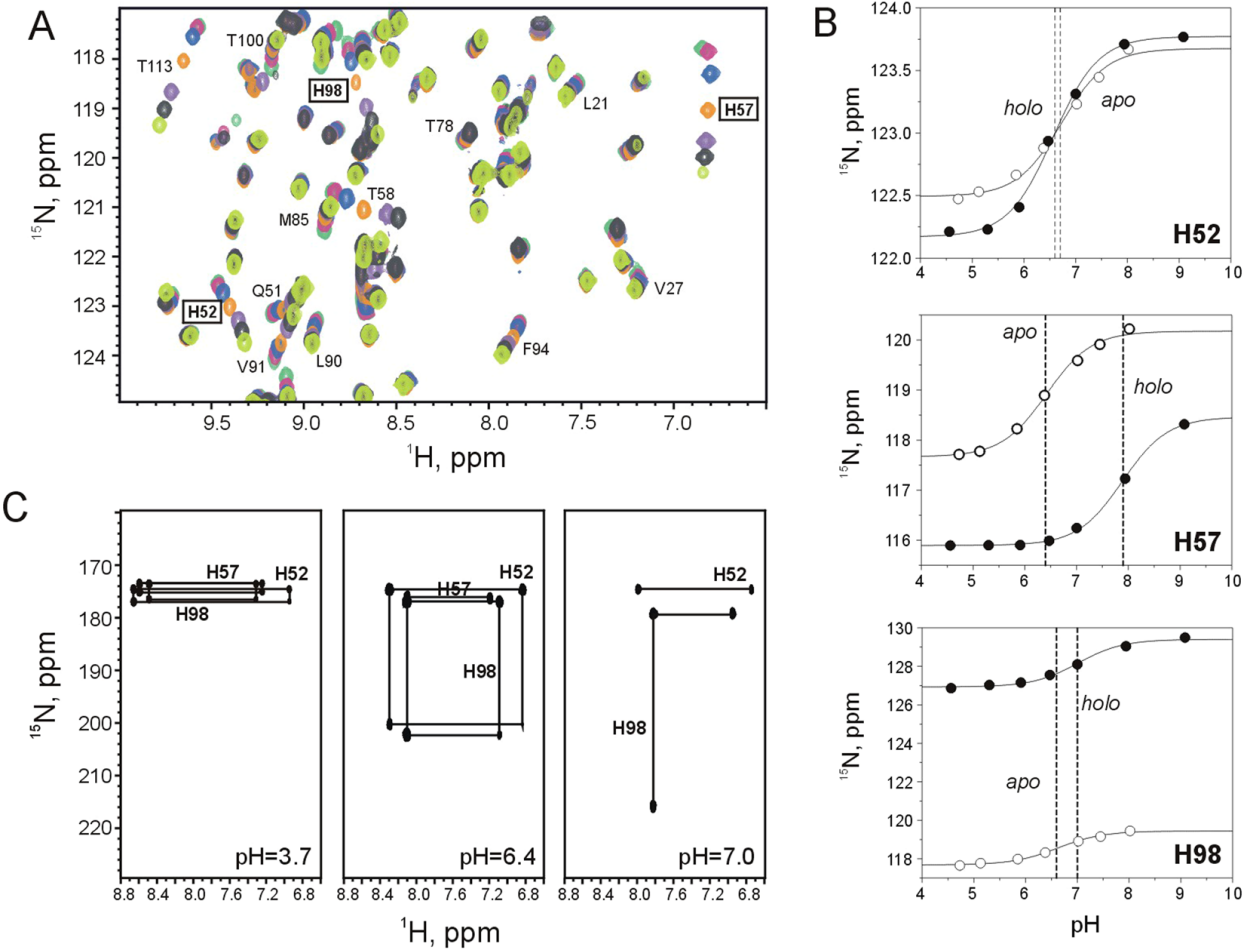
(**A**) Selected region of the ^15^N-HSQC spectrum of *apo* human I-BABP at 10 °C and pH 4.7 (dark green), 5.1 (pink), 5.9 (blue), 6.4 (orange), 7.0 (purple), 7.5 (grey), 8.0 (light green). (**B**) Titration curves for the backbone ^15^N resonance of the histidines in the *apo* (○) and the *holo* (●) states. Dashed lines show the estimated value of pK_a_ as determined from fitting the observed ^15^N chemical shifts to eq. 4. (**C**) ^1^H-^15^N long-range HSQC spectra of *apo* human I-BABP at pH 3.7 (*left*), 6.4 (*middle*), and 7.0 (*right*) (10 °C).

**Figure 5:**
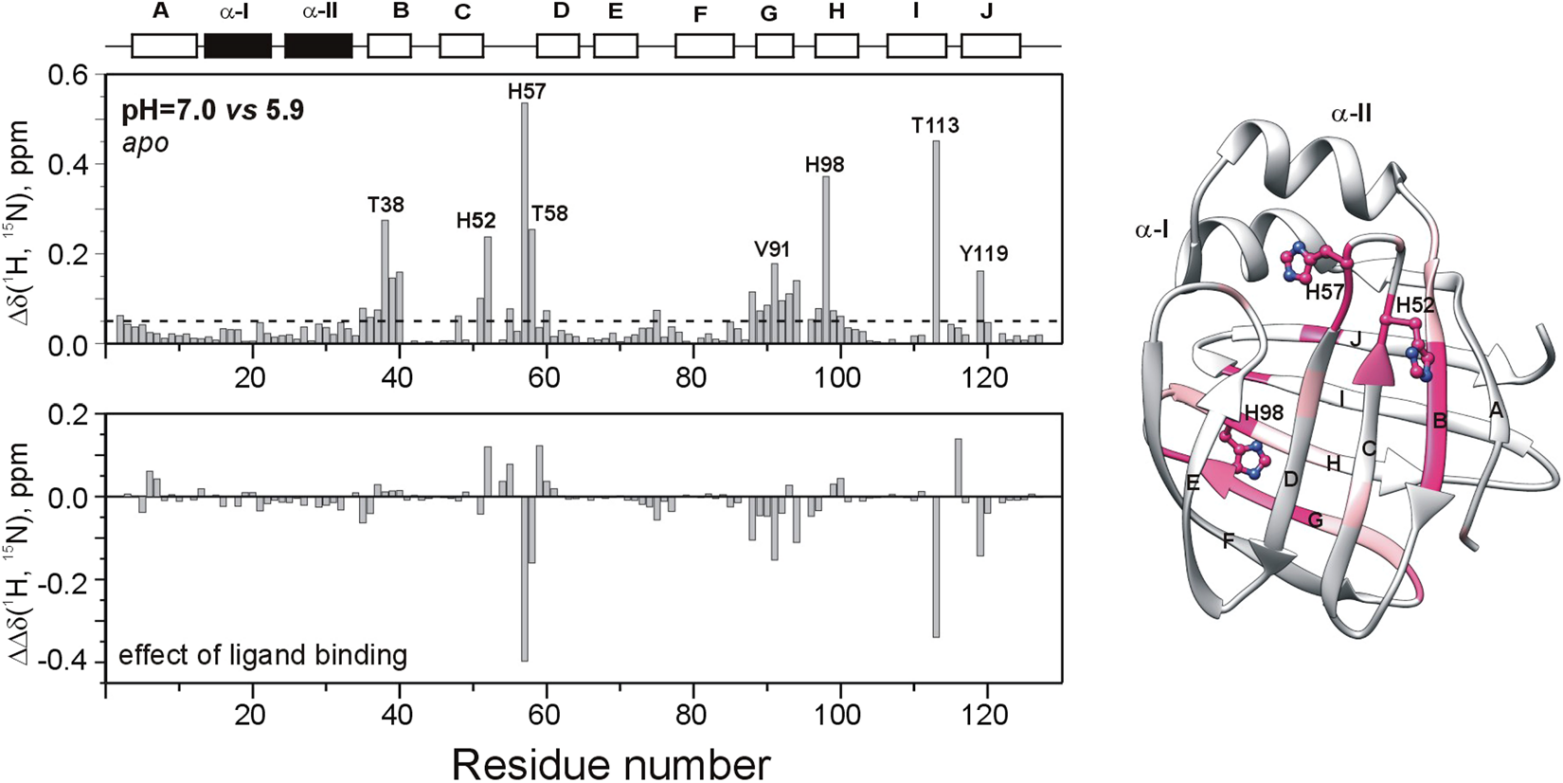
Backbone amide chemical shift differences for *apo* human I-BABP between pH = 5.9 and 7.0 (*top left*). Combined (^1^HN, ^15^N) chemical shifts were calculated using the equation of Δδ_1HN, 15N =_√[(Δδ_HN_)^2^ + (w_1_*Δδ_N_)^2^], where w_1_ (=0.154) is a weight factor determined using the BioMagResBank chemical shift database^61^. The dashed line corresponds to the mean value. Residues with substantially above average (mean + 0.5 SD and mean + SD in light and dark pink, respectively) are mapped on the ribbon diagram of hI-BABP (PDB entry 1O1U^56^). The position of the histidines is indicated in a ball-and-stick representation. The effect of ligand binding on pH-induced chemical shift changes is depicted at the bottom.

To explore the long-range effects of histidine protonation, chemical shifts have been monitored in *apo* human I-BABP and the doubly-ligated human I-BABP:GCDA:GCA (1.0:1.5:1.5) complex in the range of pH = 4.5–9.0. Superimposed partial ^1^H−^15^N-HSQC spectra of *apo* human I-BABP showing the pH-dependence of the three histidines together with some of the residues in their vicinity exhibiting a similar pH-response are depicted in Fig. 4A. Quantifiable chemical shift changes as a function of pH have been fitted using eq. 4 (Fig. 4B), yielding apparent local pK_a_ values listed in Table 3. In the *apo* state, H52, H57, and H98 titrate with a pK_a_ = 6.7, 6.4, and 6.6, respectively, which is in reasonable agreement with theoretical pK_a_ values (6.2, 6.3, and 6.3, respectively) calculated using a Gaussian-based dielectric function^25^. Upon ligand binding, the pK_a_ of H57 increases markedly to 7.9 due the presence of the negatively charged bile salt side chains in the upper segment of the binding cavity and favourable electrostatic interactions with the closing helical cap. A smaller but significant increase in pK_a_ of ~0.4 unit occurs for H98 as well upon bile salt binding, whereas the pK_a_ of H52 remains nearly unaffected. Long range effects of histidine protonation are detected in two main regions of human I-BABP (Fig. 5). pH-induced chemical shift changes in βB (T38-V40) are dominated by the effect of the electrostatic interaction between the carboxylate group of E39 and the imidazole ring of H52, whereas perturbations in βG (G88-N93), the G/H-turn (F94, N96), and part of the I/J-region (T113, Y119) reflect changes in the protonation state of H98. The latter is indicated by highly similar values of pK_a_ in the *apo* state (pK_a_ = 6.6–6.8) and a similar shift of pK_a_ upon ligation (pK_a_ = 7.0–7.2) for the affected residues (Table 3). Importantly, for most of this protein region, pronounced ^15^N chemical shift changes (0.2–0.4 ppm) suggest significant structural perturbations upon changes in H98 protonation. An H-bonding network (H98-T113, S112-Y119, Y97-F94, Q99-V92) involving the neighboring GHIJ strands appears to have an important role in the transmission of pH-coupled structural rearrangements. Long-range structural effects seem to be associated with H52 protonation as well manifested in significantly above average pH-induced ^15^N chemical shift changes nearly the entire length of βB. Additionally, as indicated by their pK_a_ in the holo state, residues at the beginning of βD (T58-T60), exhibit a pH-response similar to H52 rather than the more solvent-exposed H57.

**Table 3:** Apparent pK_a_ values of histidines and residues in their vicinity on the basis of their ^15^N chemical shift changes as a function of pH in *apo* and *holo* human I-BABP (10 °C).

Residue	*apo*	*holo*
Thr_38_	N/A	6.5
Glu_39_	N/A	6.5
Val_40_	N/A	6.8
His_52_	6.7	6.6
His_57_	6.4	7.9
Thr_58_	6.3	6.6
Met_59_	N/A	6.6
Thr_60_	6.6	6.3
Val_92_	6.7	N/A
Asn_93_	6.7	7.2
Phe_94_	6.7	N/A
His_98_	6.6	7.0
Thr_100_	N/A	7.0
Thr_113_	6.8	7.2

Changes in histidine protonation states as a function of pH have also been monitored by long-range ^15^N-HSQC experiments that correlate histidine ^15^N_δ1_ and ^15^N_ε2_ chemical shifts with nonexchangeable C-H ring protons (Fig. 4C). Based on studies of free histidine and model compounds, the protonated and deprotonated nitrogen of the neutral tautomer in either the δ_1_ or the ε_2_ position is expected to resonate at 167.5 and 249.5 ppm, respectively^26^, whereas in the cationic form the resonance frequency of ^15^N_δ1_ and ^15^Nε_2_ are at ~178 and 174 ppm, respectively^27,28^. As expected, at low pH (3.7), the protonated form dominates for each of the three histidines in *apo* human I-BABP. At pH = 6.4, H52 and H98 exist in a mixture of different forms, including the neutral Nδ_1_-H/N_ε2_ and N_δ1_/Nε_2_-H tautomers with the most abundant N_δ1_/Nε_2_-H form appearing to be more prevalent. Based on the ^15^N chemical shifts, a substantial population of the cationic form exists as well. The proton of H57 at this pH seems to be in fast exchange between N_δ1_ and Nε_2_. Further increasing the pH to 7.0 results in the typical Γ pattern of cross-peaks^29^ characteristic of the N_δ1_/Nε_2_-H neutral tautomer for H98 whereas for H52 the proton is exchanging between N_δ1_ and Nε_2_ in a fast tautomerization process. The cationic form of H52 and H98 is still present. The connectivity pattern of H57 is not detectable due to high mobility. Similar connectivity patterns were obtained for the *holo* state as well with slight perturbations in chemical shifts (data not shown).

### pH dependence of slow conformational fluctuations

**Figure 6:**
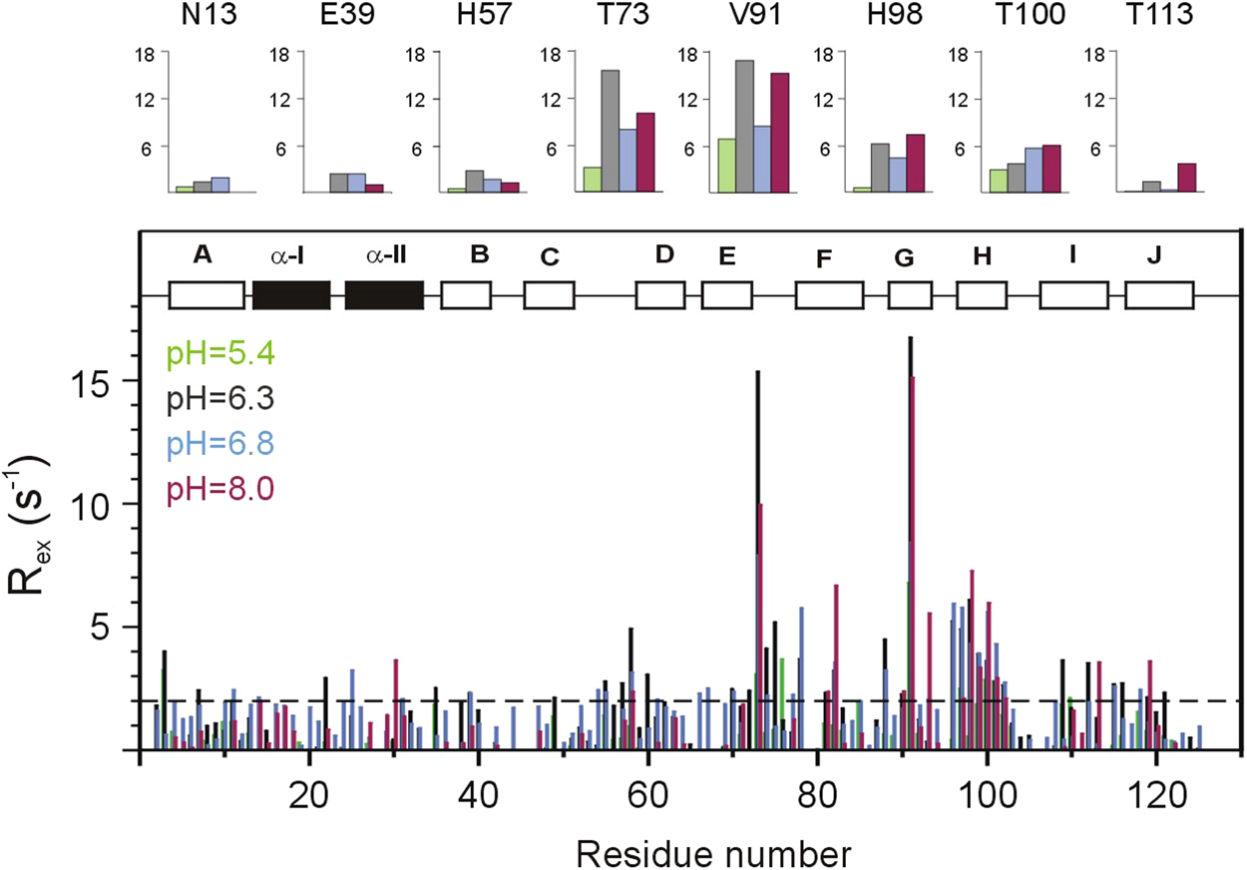
Values of ^15^N R_ex_ (at a static magnetic field strength of 14.1 T) of ^2^H/^15^N-labeled *apo* human I-BABP at 10 °C and pH = 5.4 (green), 6.3 (grey), 6.8 (blue), and 8.0 (magenta) as a function of amino acid sequence. R_ex_ was estimated from the difference in R_2eff_ at the lowest and highest ν_CPMG_ values. Secondary structural elements are indicated at the top. For representative residues, ^15^N R_ex_ at the four investigated values of pH are shown in large at the top.

**Figure 7:**
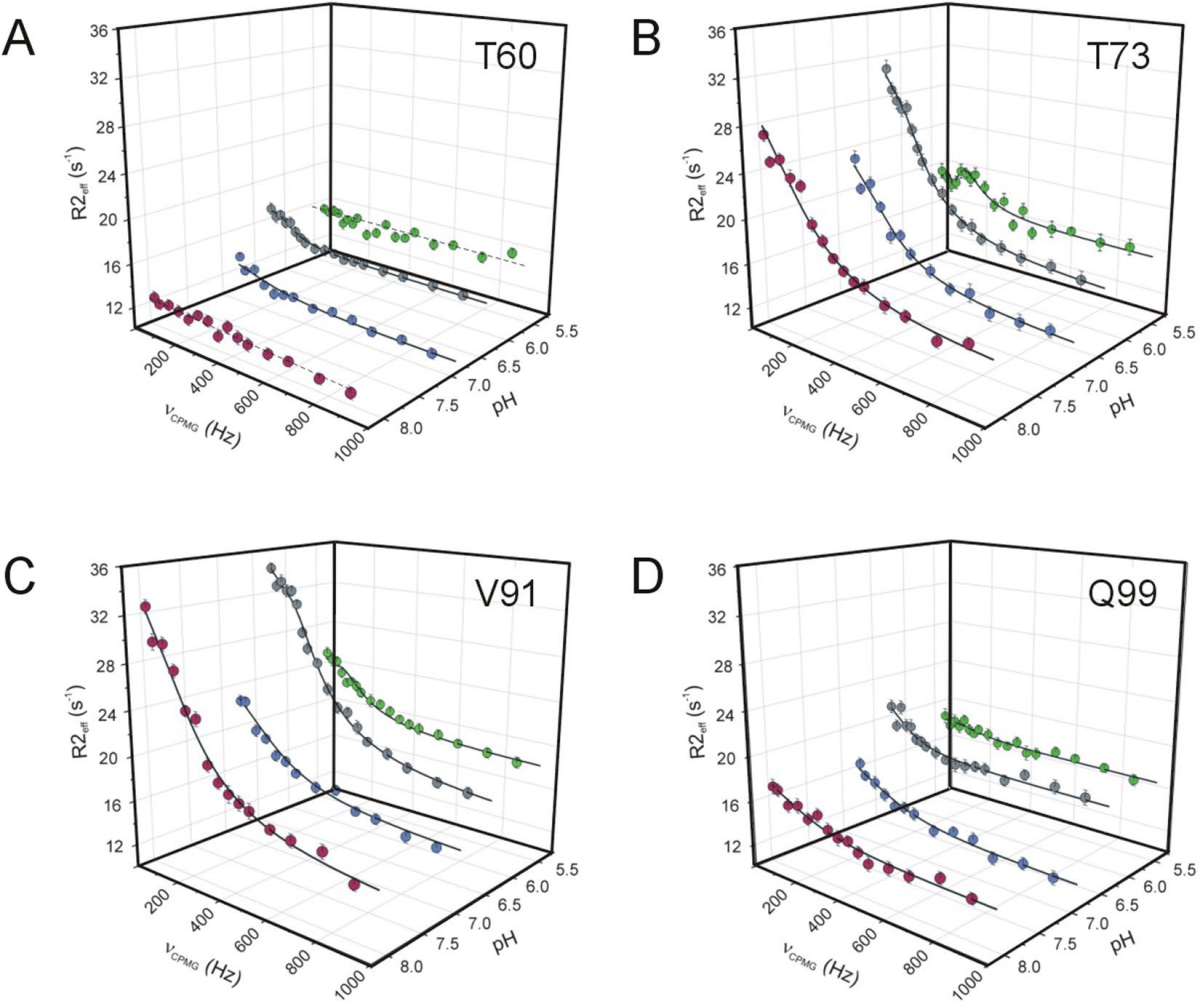
Transverse relaxation dispersions of the backbone ^15^N nuclei of selected residues in *apo* human I-BABP as a function of CPMG B_1_ field strength at 10 °C and pH = 5.4 (green), 6.3 (grey), 6.8 (blue), and 8.0 (magenta). At pH = 6.3, 6.8, and 8.0, solid black lines correspond to global two-state exchange models with parameters listed in Table 4A. Parameters providing an adequate fit for the relaxation dispersion of T73, V91, and Q99 at pH = 5.4 are listed in Table 4B. Dashed lines indicate the flatness of the dispersion profile of T60 at pH = 5.4 and 8.0.

**Figure 8:**
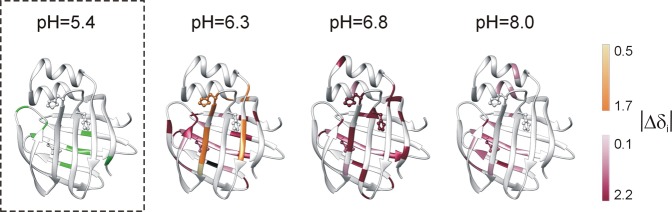
Backbone ^15^N chemical shift differences between the ground and higher energy state (|Δδ_i_|) as derived from CPMG relaxation dispersion measurements on *apo* human I-BABP at different values of pH with parameters listed in Table 4A. Values of |Δδ_i_| are mapped on the ribbon representation of the mean structure of the protein (PDB entry 1O1U^56^ in pink-to-red (pH = 6.3 ‘fast’ cluster, pH = 6.8, pH = 8.0) and yellow-to-orange (pH = 6.3 ‘slow’ cluster) gradients. Residues with non-flat R_ex_ profile at pH = 5.4 are indicated in green. Residues exhibiting a flat dispersion profile or with no available data are coloured in grey.

To relate the macroscopic thermodynamic and kinetic parameters of ligand binding to slow internal dynamics, relaxation dispersion (R_ex_) NMR measurements^30,31^ were performed on *apo* human I-BABP and the doubly-ligated human I-BABP:GCDA:GCA (1:1.5:1.5) complex at different values of pH at a static magnetic field strength of 14.1 T. Specifically, our previously obtained dataset at pH = 6.3^17^ was complemented by three additional measurement series at pH = 5.4, pH = 6.8, and pH = 8.0. As judged by the number of residues exhibiting values of R_ex_ > 2 Hz in the *apo* form, conformational fluctuations on the R_ex_-sensitive 0.3–10 ms timescale are most pronounced between pH = 6.3–6.8 (Fig. 6). Under more basic conditions (pH = 8.0), slow motions remain prevalent in the C-terminal half but become diminished in the BCD-region. The most dramatic change occurs under more acidic conditions (pH = 5.4), where the number of residues sensing a contribution to transverse relaxation from ms timescale fluctuations drops to less than 10%. Representative transverse relaxation dispersions as a function of Carr-Purcell-Meiboom-Gill (CPMG) field strength as determined for *apo* human I-BABP at the four investigated values of pH are plotted in Fig. 7. As we have described it earlier^17^, at pH = 6.3, dispersion profiles can be grouped into two clusters of residues and fit adequately with the assumption of two separate two-state exchange processes. On the basis of the slightly different exchange rate constants (k_A↔B_ = 836 ± 59 s^−1^ and k_A↔C_ = 294 ± 40 s^−1^) for the two subsets of residues, they were termed as fast and slow clusters, respectively. Importantly, a fairly good spatial separation has been observed for the two clusters with residues undergoing the faster A ↔ B process are primarily located in the E-F and G-H regions (cluster I), whereas residues involved in the slower A ↔ C process are located in β-strands B and D, the C/D-turn, and the helical cap (cluster II) (Fig. 8). Upon increasing the pH to 6.8, the exchange kinetics becomes faster and the slower cluster catches up with the faster one merging into a single fluctuating network of residues with values of k_ex_ averaging around 1316 ± 174 s^−1^. Similarly, at pH = 8.0, residues undergoing a ms timescale conformational exchange in the C-terminal half fit well to a two-state exchange model with average values of k_ex_ = 1472 ± 233 s^−1^, justifying a global fit. We note that a similar merging of distinct ms fluctuations have been observed for ribonuclease A at the pH of optimal catalytic activity^32^. As opposed, under more acidic conditions (pH = 5.4), residues with non-flat R_ex_ profile display a more heterogeneous exchange kinetics in human I-BABP with k_ex_ ranging between 12–1850 s^−1^ accompanied by large differences in the excited state populations (0.3–23%). Kinetic and thermodynamic parameters determined from the global (pH = 6.3–8.0) and individual (pH = 5.4) fit analysis are listed in Tables 4 and S1–S4 of the Supplementary Information. Besides the kinetics becoming faster, the population of the higher energy state (p_E_) increases slightly upon increasing the pH, suggesting that the conformational exchange is triggered by the formation of the deprotonated state. The majority of R_ex_-sensing residues can be characterized with a ^15^N chemical shift difference between the ground and higher energy states of |Δδ| = 0.5–2.2 ppm in the investigated pH range (Fig. 8). The exceptions include V91 exhibiting |Δδ| = 3.5 ppm at pH = 6.3 and some of the residues at pH = 5.4 (Table S1).

**Table 4:** Kinetic and thermodynamic parameters of conformational exchange in *apo* human I-BABP deduced from ^15^N backbone relaxation dispersion NMR measurements (10 °C).

*(A)*	pH = 6.3		pH = 6.8	pH = 8.0
***global fit analysis***				
	*cluster I*	*cluster II*		
k_ex_ (s^−1^)	836 ± 59	294 ± 40	1467 ± 161	1453 ± 194
p_E_ (%)	3.1 ± 0.2	1.8 ± 0.2	4.4 ± 0.3	5.7 ± 0.4
k_GE_ (s^−1^)	26 ± 3	5.3 ± 0.9	65 ± 8	83 ± 12
k_EG_ (s^−1^)	810 ± 57	289 ± 39	1402 ± 154	1370 ± 183
***(B)***	**pH = 5.4**			
***individual fit analysis***				
	**k**_**ex**_ (**s**^**−1**^)		**p**_**E**_ (**%**)	
Thr_3_	54 ± 22		8 ± 5	
Thr_73_	10 ± 44		19 ± 6	
Gly_76_	1170 ± 322		21 ± 8	
Val_91_	1852 ± 216		24 ± 3	
Tyr_97_	1057 ± 170		0.3 ± 0.4	
Thr_100_	479 ± 63		0.7 ± 0.2	
Ser_101_	651 ± 104		23 ± 5	
Glu_110_	480 ± 94		0.4 ± 0.3	

Although the changes in |Δδ| *vs*. pH (Tables S2–S4) are small and the number of data points where comparisons can be made are rather limited, a subtle difference seems to exist between the two protein regions hosting the three histidines. Specifically, a decrease in |Δδ| *vs*. pH is indicated at T60 of βD (1.5 ± 0.1 ppm at pH = 6.3 *vs*. 0.8 ± 0.1 ppm at pH = 6.8), T73 of the proximate E/F-turn (2.8 ± 0.1 ppm at pH = 6.3 *vs*. 1.7 ± 0.2 ppm at pH = 6.8 *vs*. 1.9 ± 0.2 ppm at pH = 8.0), and to a smaller extent, at E16 of the nearby α-I (0.83 ± 0.05 ppm at pH = 6.8 *vs*. 0.6 ± 0.1 ppm at pH = 8.0). This trend appears to be different from what we observe in the I/J-region of the C-terminal half, where a slight increase in |Δδ| is found at T113 (0.57 ± 0.03 ppm at pH = 6.3 *vs*. 0.9 ± 0.1 ppm at pH = 8.0) and Y119 (0.75 ± 0.03 ppm at pH = 6.3 *vs*. 1.1 ± 0.1 ppm at pH = 8.0). In the G/H-region, the observed differences are either within experimental error or the change of |Δδ| with pH appears to have a maximum (Y97, T100, S101) or a minimum (V91) at pH = 6.8, that is near the pK_a_ of H98. To confirm these trends, additional experiments are required, but the observed differences may indicate that while in the vicinity of the C/D-turn (hosting H52 and H57), the excited state may be reminiscent of a non-protonated form, in the I/J-region (proximate to H98) it may carry features of a protonated state.

In contrast to the unligated state, in *holo* human I-BABP the vast majority (>95%) of relaxation dispersion profiles remain flat in the investigated 5.4–8.0 pH range. Exceptions occur sporadically throughout the sequence and are limited to F2, Y14, S25, R32, T78, and G115 located in turn or linker regions or at the termini of secondary structural elements. Importantly, ceasing of slow motions upon ligand binding is observed in all regions exhibiting an exchange in the *apo* state. This is in agreement with our previous findings^13,17^ and indicates that ms conformational fluctuations giving rise to R_ex_ in the unligated form become abolished upon bile salt binding in a wide pH range.

### Molecular dynamics simulations

**Figure 9:**
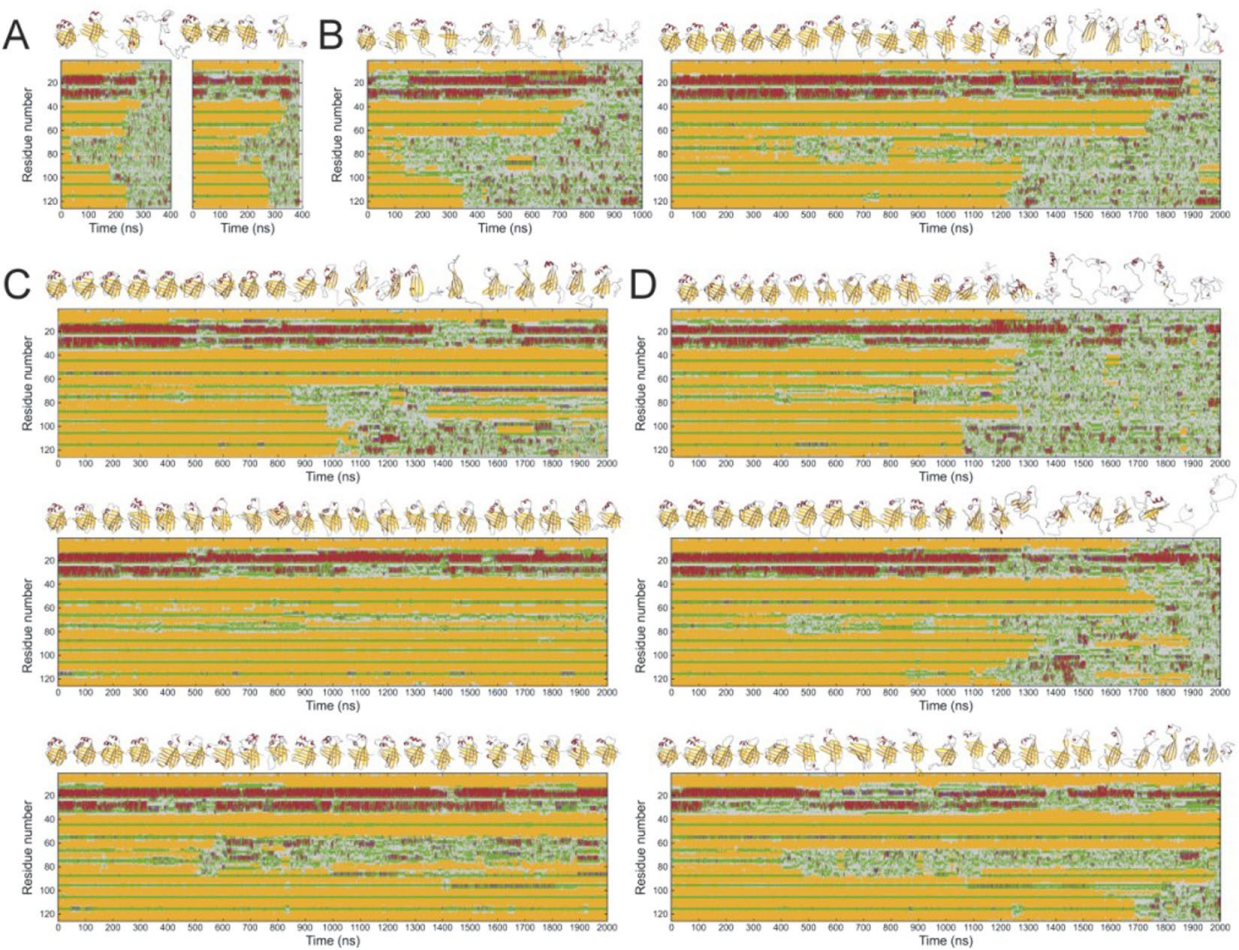
The effect of His protonation on the stability and flexibility of human I-BABP as revealed by MD simulations. (**A**,**B**) I-BABP with uncharged (left) and all cationic (right) H52, H57, and H98 residues simulated at 400 K (**A**) and 370 K (**B**). (**C**) MD at 370 K of single charged variants, with cationic H52 (top), H57 (middle) and H98 (bottom). (**D**) MD at 370 K of double charged variants, 52ε57p^+^98p^+^ (top), 52p^+^57ε98p^+^ (middle) and 52p^+^57p^+^98ε (bottom). Secondary structure composition is presented along the sequence as a function of time (colour coding: red: α-helix; purple: 3_10_-helix, yellow: β-strand, green: turn, grey: irregular). 3D structures corresponding to every 200 ns are also presented.

We tested the effect of protonation levels of the three His residues on the flexibility and stability of human I-BABP by MD simulations. First, simulations with uncharged, Nε2 protonated His residues (designated as 52ε57ε98ε) and with the fully protonated 52p^+^57p^+^98p^+^ models were carried out at 300, 350, 370, and 400 K. At 300 K and 350 K, on the short, μs time scale of the MD simulations there were no significant differences observable between the variants. At 400 K, both variants exhibited a complete unfolding during the simulation, however, the 52ε57ε98ε variant unfolded significantly faster (Fig. 9A). We found that on the time scale of these MD simulations, 370 K is the temperature where the stability of the I-BABP molecule is challenged and differences are well expressed between His protonation variants. We have found that at this temperature protonation decreases the flexibility in most regions and stabilizes the secondary structural elements in general. In the case of 52ε57ε98ε I-BABP, starting with the flexible EF strands, the C-terminal half of the molecule became unfolded after 350 ns followed by a complete unfolding in the second half of the simulation. The fully His-protonated variant 52p^+^57p^+^98p^+^ was proved to be substantially more stable, however, this variant also lost its structure completely at the end of a 2 μs simulation (Fig. 9B).

In an attempt to distinguish the role of the individual histidines in the stability and dynamics of hI-BABP, we carried out 2 μs simulations with one (Fig. 9C) and two (Fig. 9D) charged histidines in all variations. Uncharged histidines were protonated at Nε2. Comparing the α-helical region (Tyr14-Phe34) in different human I-BABP His protonation variants, it was found that the positive charge at His98 exhibited a stabilization effect increasing the average α-helix content in all but one cases (Table 5). The exception was the variant charged only at His57, which exhibited the highest of all helix content and has proven to be the most stable variant (*cf below*).

**Table 5:** The effect of His98 protonation on the alpha-helical region.

Variant	Number of residues in α-helix	Variant	Number of residues in α-helix
52ε57ε98ε	7.4	52ε57ε98p^+^	9.3
52ε57p^+^98ε	12.4	52ε57p^+^98p^+^	7.6
52p^+^57ε98ε	6.9	52p^+^57ε98p^+^	7.7
52p^+^57p^+^98ε	5.4	52p^+^57p^+^98p^+^	7.9

Average number of residues in α-helix conformation in the Tyr14-Phe34 protein region during the MD simulations at 370 K is listed for different human I-BABP variants. The calculation was limited to the part of the simulations where the region of His98 was structured. Variants with neutral and with positively charged His98 are shown in the left and right columns, respectively.

Compared to the molecule with uncharged histidines, all single charged variants proved to be more stable, however, to a different extent. The C terminal β-sheet of 52p^+^57ε98ε hI-BABP unfolded after 1 μs, while the N-terminal part was basically intact until the 2 μs end of the simulation (Fig. 9C, *top*). The 52ε57p^+^98ε variant showed the highest stability not only in single charged but among all variants with low fluctuations in the β-barrel structure (Fig. 9C, *middle)*. The 52ε57ε98p^+^ variant was stable during the 2 μs simulation, however, its DEF strands unfolded at ~550 ns, and part of the former EF region made a new strand pairing to the C strand forming a misfolded intermediate with an open, exposed substrate binding site which was stable for the rest of the simulation (Fig. 9C, *bottom*).

Double charged human I-BABP variants were proved to be stable in the first 1 μs of the simulations with showing the previously described flexibility^18^ in the region of the EF strands (Fig. 9D). However, the 52ε57p^+^98p^+^ variant became unfolded around 1200 ns. The 52p^+^57ε98p^+^ variant was more stable but its C-terminal unfolded around 1300 ns followed by a complete unfolding around 1800 ns. The most stable double charged variant was 52p^+^57p^+^98ε with uncharged His98 showing flexible α-helices (Fig. 9D, *bottom*) and EF region with a partial C-terminal unfolding at the end of the 2 μs simulation.

In summary, compared to the molecule with all neutral histidines, the cationic His residues increased the stability of human I-BABP, however, in a non-additive manner. The most stable variant was proved to be the one with single cationic His57. Protonation of His98 increased the stability of the N-terminal α-helices. With all three histidines charged, the stability of hI-BABP was in the middle among the variants. The unfolding of hI-BABP usually started with unfolding of the flexible EF strands and the C-terminal β-sheet, indicating the vulnerability of the C-terminal half and higher stability and rigidity of the N-terminal β-sheet (A-D strands) of the molecule.

## Discussion

Bile salts are amphipathic molecules synthesized from cholesterol in the liver, which in the small intestine facilitate the absorption of dietary lipids, cholesterol, and fat-soluble vitamins. Besides their role in digestion, they activate signaling pathways^33,34^, nuclear hormone receptors^35,36^, and have been shown to interact with the G-protein coupled receptor TGR5^37,38^. Participating in a diverse set of activation processes, bile acid-controlled signaling pathways are important new targets for the treatment of metabolic disorders^39^. Human I-BABP binds and carries bile salts in the epithelial cells of the distal small intestine and has a key role in the enterohepatic circulation of bile salts^40^. Additionally, BABPs are thought to have a role in gene regulation by controlling the presentation of bile salts to the farnesoid X receptor (FXR)^35^.

Positive binding cooperativity between the two binding sites in combination with their relatively low intrinsic affinity enables human I-BABP to act as a buffering agent, which at low bile salt concentration allows a sizeable fraction of bile salts to remain unbound (passing through the enterocytes as monomers) whereas at high bile salt concentrations it protects cells from toxicity^20^. The extensive hydrogen bond network mediating the communication between the two binding sites^21^ in conjunction with large conformational rearrangements observed upon ligand binding in the G/H- and C/D-turn regions^6^ hosting the three histidines of the protein prompted us to investigate the effect of pH on the binding process and internal dynamics of human I-BABP.

One of the most important findings of our work is the overlap between residues exhibiting an above average sensitivity to pH change in their backbone ^1^H/^15^N chemical shifts and protein regions undergoing a conformational exchange on the fast end of the millisecond timescale in *apo* hI-BABP. As indicated by our NMR relaxation measurements, the contribution of the motion to transverse relaxation is most pronounced between pH = 6.3–6.8, that is near the pK_a_ of the histidines of hI-BABP (Table 3). Under more basic conditions (pH = 8.0), while ms timescale fluctuations in the C-terminal half still persist (with exchange rate constants and populations similar to those observed at pH = 6.8 but with somewhat smaller values of R_ex_), conformational exchange in the vicinity of the C/D-turn becomes abolished. Regarding the opposite, i. e. under conditions corresponding to each of the histidines being protonated (pH = 5.4), fluctuations on the ms timescale become markedly diminished and more heterogeneous by means of both the kinetics and local thermodynamics of the exchange. The cessation of motion below the pK_a_ of histidines is in agreement with MD simulations showing a decrease in flexibility and enhanced stability of the 52p^+^57p^+^98p^+^ state in comparison to 52ε57ε98ε.

When comparing the ^15^N chemical shift differences between the ground and the excited states inferred from the R_ex_ measurements (|Δδ|) with differences between chemical shifts obtained under more acidic and more basic conditions, most R_ex_-sensing residues exhibit a value of |Δδ| exceeding the pH-induced shift change. Accordingly, while the observed conformational fluctuation appears to be associated with the protonation/deprotonation equilibrium of histidines, the resulting conformational change is more complex manifesting in larger changes in the chemical environment of the affected residues. As we have shown previously, the slow conformational fluctuations in the *apo* state near neutral pH cease upon bile salt binding and the R_ex_-detected higher energy state in the free form shows similarities with the *holo* state^17^. Specifically, the correlation between the R_ex_-derived values of |Δδ| and ^15^N chemical shift differences between the *apo* and *holo* states for a subset of residues in the C-terminal half (e.g. I71, M74, T78, G88 in the EF- and V91, Q99, S101 in the GH-region) suggests the presence of an equilibrium between a closed and a more open *holo*-like state in the absence of ligands. According to the conformational selection mechanism we proposed earlier^6^, the closed state is thermodynamically favoured in the absence of ligands, whereas upon bile salt binding the equilibrium is shifted toward the open state possessing an enlarged gap between the E/F- and G/H-turns. More recently, as revealed by temperature dependent NMR measurements and MD simulations^18^, ms timescale fluctuations in the C-terminal half are related to a partial unfolding of hI-BABP in the DEF protein region. Specifically, a joint analysis of thermal melting and relaxation dispersion data at pH = 6.3 reveals a correlation between R_ex_-derived Φ values and the squared chemical shift difference between the folded and a partially unfolded hI-BABP state deconvoluted from NMR thermal melting curves indicating a direct connection between the two processes in the *apo* form^18^. This raises a possibility that the excited state carries partially unfolded characteristics in the C-terminal half with an opening in the EFGH region, which becomes stabilized upon ligand binding. We note that the opening in the EFGH region is accompanied by a closure of the helical cap on the β-barrel by both NMR structural studies (*holo vs*. *apo* form)^6^ and MD simulations^18^. A dynamic connection between the G/H-region and the helical cap is further indicated by MD simulations in the current study suggesting that the protonation state of H98 has a role in the stability of the helical region (Table 5). The MD simulations also highlight the role of H57 in the stability of the protein suggesting that favourable interactions of the protonated H57 with negatively charged side chains of the helical cap (i. e. D26) may trigger a chain of stabilizing interactions propagating from the C/D-turn and the helices to the C-terminal half of the protein with the capability of enhancing the stability of even the loose and vulnerable DEF-strands as well (Fig. 9C, *middle*). Intriguingly, while protonation of H98 in itself has a long-range stabilizing effect on the helical region, protonation of H98 together with either one or both of the histidines in the C/D-turn appears to be less favourable for both the helical content (Table 5) and the overall stability of the protein (Fig. 9D). One possible explanation for this is that positively charged histidines in both regions interfere with some of the hydrophobic interactions stabilizing the protein scaffold.

**Figure 10:**
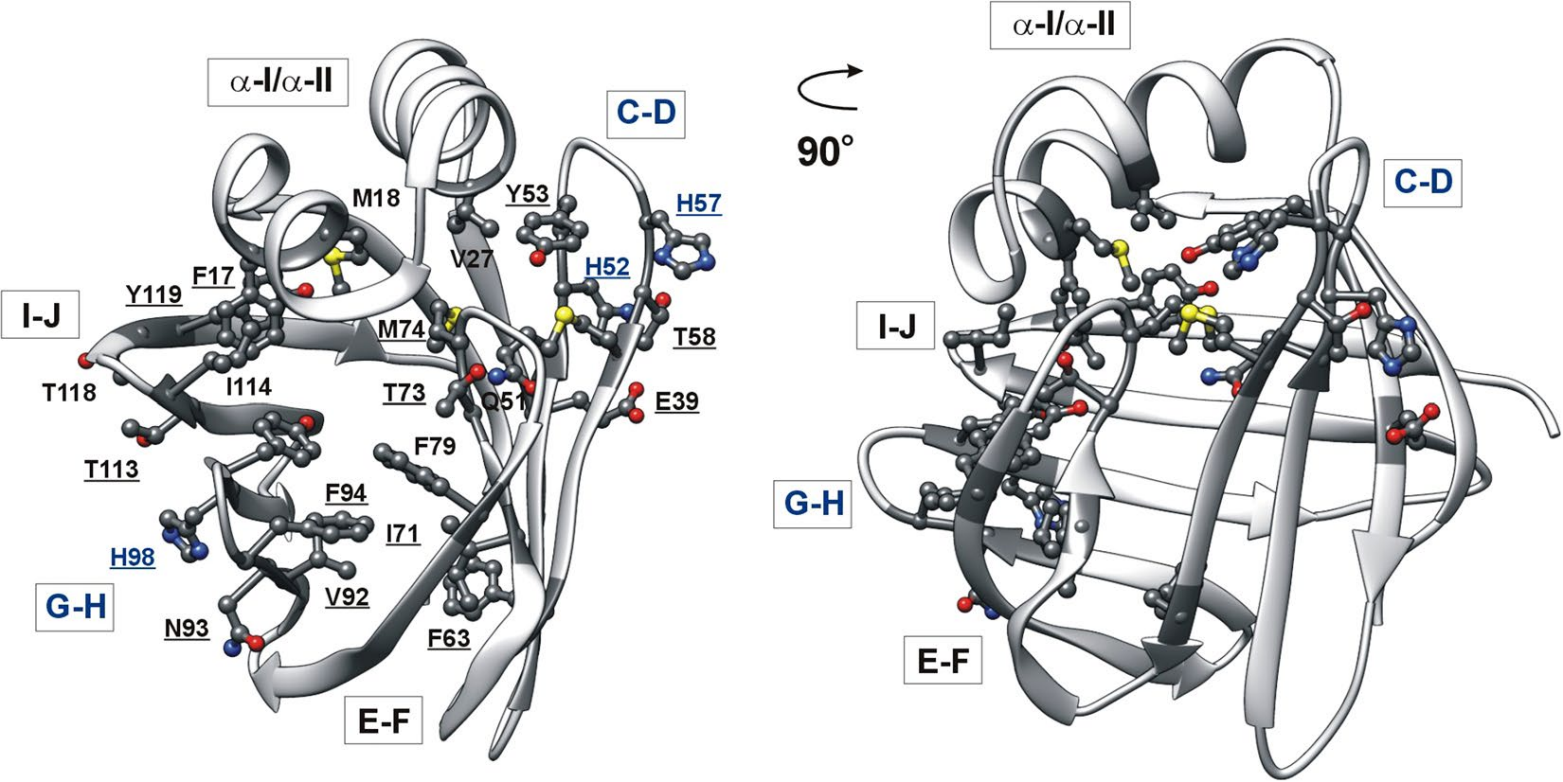
Stabilizing interactions in the doubly-ligated hI-BABP:GCDA:GCA complex (PDB entry 2MM3^6^) corresponding to the ‘EFGH-open/helix-closed’ state of the protein. Hydrogen-bonding and hydrophobic interactions mediate the flow of information between distant sites including βH and the C/D-turn hosting the three histidines and segments in the EF-, IJ-, and helical-regions. Residues with non-flat R_ex_ profile at pH = 6.3 (10–18 °C) are underlined.

Cessation of the slow fluctuation upon either bile salt binding or lowering the pH in the *apo* form indicates similarities in the dynamic behaviour of the complexed and the histidine-protonated *apo* forms of the protein. One possibility is that similarly to ligand binding, histidine protonation stabilizes a more open arrangement of the EFGH β-strands in the putative portal region. As we have shown previously for the *holo* protein^6^, a more open conformation in the EFGH region can be stabilized by the formation of new hydrogen-bonds with the involvement of H98 and its vicinity (e.g. N_δ1_ of H98 and O_δ1_ of N93, OH of Y97 and O_ε1_ of Q99, NH of N96 and CO of G115, OH of T113 and OH of T118, NH of T113 and CO of H98) as well as the rearrangement of hydrophobic interactions between the D and E β-strands (e.g. F63-I71, M59-M74) (Fig. 10). It is also apparent that the accompanying closure of the helical cap results in contacts between the I-J region (I114, Y119) and α-I (F17, M18) as well as between the helical segment (M18, D26, V27) and the C/D- (Y53, H57, M59) and E/F-turn regions (M74). Accordingly, an extensive network of interactions can form in the ‘EFGH-open/helix-closed’ state enabling the transmission of information from the segments hosting the histidines to the IJ-, EF-, and the helical regions (Fig. 10). In agreement with this, MD simulations show the involvement of the C/D-turn and D beta-strand in the correlated ‘breathing’ motion of human I-BABP indicating that the motions in the C-terminal half (EFGH-region) and the CD-region with the together moving helical cap are closely related^18^. Noteworthy, while the protonation equilibrium of H98 appears to have a dominating role in triggering the fluctuation between the closed and more open states throughout the protein, there is a pH (6.3) where the motion of the EFGH and BCD-αIαII protein regions is decoupled from each other giving rise to a faster and slower cluster of residues in the C- and N-terminal half, respectively. This suggests that the protonation of histidine(s) in the C/D-turn may contribute and fine tune the allosteric regulation of ligand entry in hI-BABP.

We attribute the differences in the dynamic behaviour between the two protein regions (i. e. ceasing of motion in the C-D region at pH = 8.0) to differences between the H98 *vs*. H52/H57 interaction networks. For instance, an H-bond/salt bridge with the proximate side chain of E39 can have an important role in shifting the protonation equilibrium of H52. Importantly, an additional H-bond with V37 further ties H52 to the neighboring B strand. Furthermore, the vicinity of H52 is tied strongly to βD by multiple H-bonds (Q51-M59, Y53-H57). This may explain the similar dynamic behaviour of βB, βD, and the C/D-turn as revealed by R_ex_-measurements in a wide temperature range^17^. Unlike the two histidines in the C/D-turn, H98 in βH forms backbone H-bonds with N93 and T113 contributing to the stabilization of the protein scaffold in the GHI-region by strand-strand interactions.

The shift of the pK_a_ of H98 and residues interacting with it to larger values upon ligand binding is consistent with the notion that bile salt binding is favoured by H98 protonation. This may provide an explanation for the faster association rate constant of bile salt binding below the pK_a_ of H98 as a shift in the conformational equilibrium toward the state with a more open conformation in the EFGH-region is expected to aid the entry of bile salts. We note that as the pK_a_ of bile salts (at least in the unbound state) is ~4, a fraction of them (<10%) may be in the protonated neutral state at the lowest investigated values of pH, which may slightly influence the binding affinity. However, as revealed by structural studies of hI-BABP (PDB: 2MM3), the carboxyl group of GCDA is in favourable electrostatic interaction with the positively charged side chain of K77 and in the vicinity of a number of polar side chains. Therefore, it is unlikely that a protonated GCDA would bind with a higher affinity. In the case of GCA, its carboxyl group is in the vicinity of K30 and H57 (the latter showing a dramatic increase in its pK_a_ upon ligand binding from pK_a_ = 6.4 (*apo*) to 7.9 (*holo*), that is favouring a protonated side chain in the presence of bile salts), therefore we find it unlikely that an uncharged GCA with a protonated carboxyl group would bind with higher affinity.

The relation of the protonation equilibrium of the conserved H98 to an open/closed conformational change has been indicated for the chicken liver BABP analogue as well^12^ suggesting that it may be a common means of an allosterically regulated binding mechanism in the iLBP subfamily. Importantly, in cL-BABP there is no histidine in the C/D-region and the interaction network appears to be more limited. (We note that there is a second more exposed histidine in cL-BABP at the bottom of the beta-barrel, which reportedly is not involved in the conformational fluctuation and forms no contacts with the bound bile salts.) Strikingly, there is a significant difference between the two proteins in terms of both the pK_a_ of H98 and its change upon bile salt binding. Unlike in the human form (Table 3), the conserved H98 in the chicken liver analogue is more buried and exhibits a pK_a_ of 4.7 in the *apo* state, which upon bile salt binding shifts further down^41^. Accordingly, while in the chicken liver analogue the contribution of H98 toward substrate binding is favoured by an increase in pH, in the human ileal form it appears to be the opposite. The difference in the values of pK_a_ indicates a large difference in the fraction of protonated H98 at physiological pH between cL-BABP and hI-BABP. Given the hypothesized role of the protonation/deprotonation triggered conformational transition in ligand entry in both analogues, the difference in histidine pK_a_ values should have implications for the regulation of free and bound bile salt pool in (human) enterocytes and (chicken) hepatocytes. Noteworthy, in chicken *ileal* bile acid-binding protein (cI-BABP), there is an additional histidine in the C/D-turn similar to the human form^42^. Also, unlike in the liver analogue, in cI-BABP, H98 is solvent exposed similar to hI-BABP.

Importantly, in cL-BABP, H98 has also been suggested to be involved in a network of communication between the two binding sites. Specifically, a hydrophobic bridge between the deprotonated H98 and the nearby I111 has been indicated to provide a connection between the two bound bile salts and contribute to binding cooperativity^24^. As noted above, in the human ileal form, the side chain of H98 is facing the solvent and does not form a contact with the bound bile salts. In agreement with this, our ITC analysis have shown no significant effect of pH on binding cooperativity in human I-BABP between pH = 5.8 and 7.2. Comparison of the bound state of cL-^41^ and hI-BABP^6^ suggests that the concerted fluctuation of the CD-turn with the EFGH-region in the human form may have a role in enabling one of the bile salts to enter more deeply into the binding cavity instead of being trapped near the helical cap. Interestingly, in the chicken ileal form, an additional histidine next to H98 has been suggested to exert too much rigidity on the system and thought to be associated with the lack of positive binding cooperativity in the protein^42^.

The pH-triggered conformational fluctuation between the closed and more open EFGH protein region in human I-BABP has implications for bile salt uptake and targeted delivery in the epithelial cells. Specifically, enterohepatic circulation facilitated by human I-BABP involves a vectorial transport of bile salts between the apical and basolateral membrane of enterocytes^40,43^. While the mechanism of bile salt uptake through the membrane is not yet clear, human I-BABP has been shown to be colocalized and functionally associated with the apical sodium dependent bile acid transporter (ASBT) on the apical membrane of enterocytes^5^. Intriguingly, VPP-c, a subunit of a vacuolar proton pump, has been identified as an interacting partner of ASBT^44^. If bile salt entry to the enclosed binding cavity of human I-BABP is regulated by a protonation/deprotonation triggered mechanism communicated via the histidines, fluctuations in pH brought about by the vacuolar H^+^-ATPase at the apical membrane of enterocytes may have a mediatory role.

In conclusion, while histidine protonation appears to be a common means of allosteric regulation in BABP proteins, with histidines in both the C/D and G/H turn regions, human I-BABP exhibits a complex regulation of the opening/closing equilibrium mediating bile salt entry. Considering the differences in the surface accessibility and interaction network of histidines among different BABP analogues suggests that fine tuning of conformational fluctuations by protonation equilibria in different organisms and tissues may have changed in accordance with the evolving pattern of H-bonding and hydrophobic interactions dictated by the local bile salt pool.

## Materials and Methods

### Sample preparation

The methods used for the expression and purification of unenriched and ^15^N- as well as ^2^H/^15^N-labeled human I-BABP used in the experiments are detailed elsewhere^13^. In the case of the NMR spectroscopic analysis of the *holo* sample, protein was complexed with an equimolar mixture of GCA and GCDA at a molar ratio of I-BABP:GCDA:GCA = 1.0:1.5:1.5, ensuring that over 99.9% of the protein was in its bound state^20^. Protein concentration was determined by absorbance at 280 nm using an extinction coefficient of 12930 M^−1^ cm^−1^ obtained by composition analysis^45^.

### Isothermal titration calorimetry

Bile salts were obtained from Sigma and were dissolved in a buffer containing 20 mM potassium phosphate, 135 mM KCl, 10 mM NaCl, 0.05% NaN_3_ at pH = 5.8 or 7.2. Protein was dialyzed into the same buffer at each pH. The calorimetry experiments were performed using a MicroCal iTC200 isothermal titration calorimeter at 25 °C. Eight injections of 0.4 μL aliquots were followed by 37 injections of 0.8 μL aliquots of 5.1 mM bile salt into the reaction cell containing 0.2019 mL of 0.2 mM human I-BABP, unless noted otherwise. Each titration series was repeated three or four times. The heats of injection were corrected for the heat of dilution of the ligand into buffer and normalized to the amount of bile salt injected. The integrated peak intensities were fit to a stepwise binding model shown schematically as

$$P+L\leftrightarrow PL\,{K}_{1}^{obs}$$

1 $$PL+L\leftrightarrow P{L}_{2}\,{K}_{2}^{obs}$$

using Bayesian analysis as described elsewhere^46^.

### Stopped-flow fluorescence spectroscopy

Kinetic measurements of bile salt binding to human I-BABP were performed using a BioLogic SFM 300 stopped-flow spectrophotometer. Ligand binding was monitored by observing the change in fluorescence using an excitation wavelength of 290 nm (4-nm bandwidth) and a 320-nm long-pass filter. Experiments were performed at 15 °C in 20 mM potassium phosphate, 135 mM KCl, 10 mM NaCl, 0.05% NaN_3_, at pH = 5.8 and 7.2. The protein, at a concentration of 2 μM, was mixed with an equal volume of GCA or GCDA in the same buffer so that the final bile salt concentrations ranged from 20 μM to 500 μM. At and below 500 μM, no self-association of bile salts was observed on the basis of light scattering. A total of 8000 points were collected in each trace. Usually, 12–14 individual traces were averaged at each set of conditions. The kinetic curves were fitted to extract the apparent rates and amplitudes using a nonlinear least-squares algorithm, with a single or a sum of exponential functions defined as

2 $$F(t)={F}_{\infty }+\sum _{i=1}^{n}{A}_{i}\exp (\,-{\zeta }_{i}t)$$

where F(t) is the fluorescence intensity at time t, F(∞) is the fluorescence intensity at t = ∞, ζ_i_ is the apparent rate of the *i*th kinetic process occurring with an amplitude A_i_. Kinetic simulations and all further analysis of the data were performed with Dynafit^47^ and Mathematica (Wolfram, Urbana, IL) assuming a four-step mechanism of binding according to

$$P\text{'}\mathop{\leftrightarrow }\limits^{{k}_{1/-1}}P$$

$$P+L\mathop{\leftrightarrow }\limits^{{k}_{2/-2}}PL$$

$$PL+L\mathop{\leftrightarrow }\limits^{{k}_{3/-3}}P{L}_{2}$$

3 $$P{L}_{2}\mathop{\leftrightarrow }\limits^{{k}_{4/-4}}P{L}_{2}^{\ast }$$

as described in detail previously^9^.

### NMR data collection and analysis

Multidimensional NMR experiments were carried out at 283 K on 600 MHz Varian NMR SYSTEM™ spectrometer equipped with a 5-mm indirect detection triple ^1^H^13^C^15^N resonance z-axis gradient probe. The backbone resonance assignment of *apo* human I-BABP and the doubly-ligated hI-BABP:GCDA:GCA complex under our experimental conditions at pH = 6.3 has been published earlier^13^. To monitor the effect of pH on backbone amide chemical shifts, a series of ^1^H-^15^N HSQC spectra^48^ were acquired on [U-^15^N]-enriched *apo* and *holo* human I-BABP in a buffer containing 20 mM potassium phosphate, 50 mM KCl, and 0.05% NaN_3_ at pH = 4.7, 5.1, 5.9, 6.4, 7.0, 7.5, 8.0 and pH = 4.6, 5.3, 5.9, 6.5, 7.0, 7.9, 9.1, respectively. Protein concentration was 0.5 mM in all experiments. To evaluate the pK_a_ values of histidines and residues in their vicinity, the observed ^15^N chemical shifts were fitted to the following equation^49^

4 $${\delta }_{15N}^{obs}={\delta }_{d}+\frac{{\delta }_{p}-{\delta }_{d}}{1+{10}^{(pH-pKa)}}$$

where δ_p_ and δ_d_ are the chemical shifts of the protonated and deprotonated forms, respectively. The protonation and tautomeric state of the histidines was further monitored by long-range ^1^H-^15^N HSQC measurements allowing the correlation of histidine ^15^N_δ1_ and ^15^N_ε2_ chemical shifts with nonexchangeable C-H ring protons. The delay to refocus ^1^H-^15^N correlations during the formation of ^1^H-^15^N antiphase magnetization was set to 23 ms. Experiments were performed at pH = 3.7, 6.4, 7.0 and 4.6, 6.5, 7.9 for *apo* and *holo* human I-BABP, respectively. Spectral processing, computer-assisted spin-system analysis, and resonance assignment was carried out using Felix 2004 (Accelrys, Inc.) and CCPNMR.

Relaxation dispersion data were obtained on [80% ^2^H, 99% ^15^N]-labeled protein at 283 K and pH = 5.4, 6.3, 6.8, and 8.0, using a relaxation compensated Carr-Purcell-Meiboom-Gill (CPMG) dispersion experiment performed in a constant time manner^50,51^. The constant time delay (T_CP_) was set to 40 ms. Spectra were collected as a series of 19 two-dimensional data sets with CPMG field strengths (υ_CPMG_) of 25, 50, 74, 99, 123, 147, 172, 195, 219, 242, 289, 335, 380, 425, 469, 556, 641, 764, and 883 Hz. A reference spectrum was obtained by omitting the CPMG period in the pulse sequence^52^. Spectra (3 s interscan delay, 24 transients) were acquired in duplicate. Contributions to transverse relaxation rates of conformational exchange were analyzed assuming a two-state exchange process using the all-timescales multiple quantum Carver-Richards-Jones formulation^53^ implemented in GUARDD^54^.

### MD simulations

Molecular dynamics simulations were carried out as it was described earlier^18^ using the GROMACS package^55^ on the solution structure of human I-BABP (PDB: 1O1U^56^) using the AMBER-ff99SB*-ILDNP force field^57^ and TIP4P parametrization^58^. The total charge of the system was neutralized, and the physiological salt concentration was set by placing Na^+^ and Cl^−^ ions. Energy minimization of starting structures was followed by sequential relaxation of constraints on protein atoms in three steps and an additional NVT step (all of 200 ps) to stabilize pressure. 1 or 2 μs trajectories of NPT simulations at 300, 350, or 370 K at 1 bar were recorded (collecting snapshots at every 20 ps). Secondary structure compositions of the frames of MD trajectories were determined by DSSP algorithm^59^. As partial protonation of histidines, which is realized in real solution, was not possible in these simulations, we compared neutral (Nε2) and cationic histidines in all combinations. Specifically, a series of simulations were carried out by varying the protonation levels of the three histidine residues, H52, H57, and H98, setting them individually as protonated at Nε2, or Nδ1, or at both nitrogens (positively charged form). Molecular graphics was performed with the UCSF CHIMERA package (University of California, San Francisco)^60^.

## Supplementary information

Ligand entry in human ileal bile acid-binding protein is mediated by histidine protonation

## Data Availability

All data generated or analysed during this study are included in the published article and its Supplementary Information File.
