# Supplementary material for: Ligand entry in human ileal bile acid-binding protein is mediated by histidine protonation
Source: Sci Rep. 2019 Mar 18;9:4825. doi: 10.1038/s41598-019-41180-7 (PMC6423008; doi:10.1038/s41598-019-41180-7)
Supplement: Supplementary file 1 — Ligand entry in human ileal bile acid-binding protein is mediated by histidine protonation [file 41598_2019_41180_MOESM1_ESM.docx]

**SUPPLEMENTARY INFORMATION**

**Ligand entry in human ileal bile acid-binding protein is mediated**

**by histidine protonation**

*Gergő Horváth^1^, Orsolya Egyed^1^, Changguo Tang^2^, Mihály Kovács^3^, András Micsonai^4^, József Kardos^4^, and Orsolya Toke^1,*^*

1 Laboratory for NMR Spectroscopy, Research Centre for Natural Sciences, Hungarian Academy of Sciences, 2 Magyar tudósok körútja, H-1117 Budapest, Hungary

2 Department of Biochemistry and Molecular Biophysics, Washington University School of Medicine, 660 South Euclid Avenue, St. Louis, Missouri, 63110, U.S.A.

3 Department of Biochemistry, ELTE-MTA “Momentum” Motor Enzymology Research Group, Eötvös Loránd University, Pázmány Péter sétány 1/C, H-1117 Budapest, Hungary

4 Department of Biochemistry, MTA-ELTE NAP B Neuroimmunology Research Group, Institute of Biology, Eötvös Loránd University, Pázmány Péter sétány 1/C, H-1117 Budapest, Hungary

**Corresponding Author**

*O. Toke, Ph.D.

Phone: +36-1-382-6575
E-mail: [toke.orsolya@ttk.mta.hu](mailto:toke.orsolya@ttk.mta.hu).

**Table S1** Exchange parameters derived from individual fit of ^15^N relaxation dispersion curves obtained for [80% ^2^H, 99% ^15^N-labeled] *apo* human I-BABP in 20 mM K-phosphate, 50 mM KCl, 0.05% NaN_3_, 10% D_2_O, pH=5.4 at 283 K.

| Residue | k_ex_ (s^-1^) | p_b_ (%) | \|Δω\| (Hz) | R_ex_ (Hz) | Φ (Hz^2^) |
| --- | --- | --- | --- | --- | --- |
| T3 | 54±22 | 8±5 | 73±14 | 4.4±0.4 | 405±277 |
| T73 | 10±44 | 19±6 | 232±48 | 2.9±0.8 | 8284±3619 |
| G76 | 1170±322 | 21±8 | 25±17 | 3.4±0.6 | 106±110 |
| V91 | 1852±216 | 24±3 | 44±5 | 7.4±0.5 | 358±74 |
| Y97 | 1057±170 | 0.3±0.4 | 195±29 | 2.0±0.6 | 127±99 |
| T100 | 479±63 | 0.7±0.2 | 159±19 | 2.6±0.3 | 172±57 |
| S101 | 651±104 | 23±5 | 18±5 | 3.2±0.3 | 57±26 |
| E110 | 480±94 | 0.4±0.3 | 171±21 | 1.5±0.3 | 113±87 |

**Table S2** Exchange parameters derived from global fit of ^15^N relaxation dispersion curves obtained for [80% ^2^H, 99% ^15^N-labeled] *apo* human I-BABP in 20 mM K-phosphate, 50 mM KCl, 0.05% NaN_3_, 10% D_2_O, pH=6.3 at 283 K.

| Residue | \|Δω\| (Hz) | R_ex_ (Hz) | *Φ (Hz^2^*) |
| --- | --- | --- | --- |
|  | **‘slow’ cluster k_ex_ = 294 ± 40 s^-1^ p_b_ = 1.8 ± 0.2 %** | | |
| E11 | 47±4 | 2.7±0.2 | 42±7 |
| N13 | 34±4 | 1.9±0.3 | 23±5 |
| K35 | 55±4 | 3.1±0.2 | 60±9 |
| T38 | 40±4 | 2.2±0.2 | 30±5 |
| E39 | 45±4 | 2.6±0.2 | 39±7 |
| V40 | 43±4 | 2.5±0.2 | 36±6 |
| H57 | 60±5 | 3.3±0.2 | 70±11 |
| T58 | 97±9 | 4.2±0.2 | 183±31 |
| T60 | 89±8 | 4.1±0.2 | 154±26 |
| N61 | 38±4 | 2.2±0.2 | 29±5 |
| K62 | 44±4 | 2.5±0.2 | 38±6 |
| F63 | 39±4 | 2.2±0.3 | 29±5 |
|  | **‘fast’ cluster’ k_ex_ = 836 ± 59 s^-1^ p_b_ = 3.1 ± 0.2 %** | | |
| E7 | 47±2 | 2.6±0.2 | 65±6 |
| I71 | 39±3 | 1.8±0.1 | 45±6 |
| Q72 | 42±2 | 2.1±0.2 | 52±5 |
| T73 | 170±5 | 15.8±0.1 | 857±66 |
| M74 | 65±4 | 4.7±0.2 | 126±14 |
| T78 | 59±3 | 4.0±0.1 | 105±10 |
| A81 | 49±4 | 2.9±0.2 | 72±10 |
| T82 | 56±3 | 3.7±0.2 | 94±9 |
| G88 | 67±5 | 4.9±0.1 | 133±16 |
| L90 | 43±4 | 2.2±0.2 | 54±8 |
| V91 | 210±10 | 18.2±0.1 | 1302±121 |
| N96 | 86±6 | 7.2±0.1 | 219±26 |
| Y97 | 67±4 | 4.9±0.1 | 133±14 |
| H98 | 84±7 | 7.0±0.1 | 211±28 |
| Q99 | 63±5 | 4.5±0.2 | 119±15 |
| T100 | 60±6 | 4.1±0.1 | 108±17 |
| S101 | 49±5 | 2.9±0.2 | 73±12 |
| E102 | 47±4 | 2.6±0.2 | 66±9 |
| V109 | 53±7 | 3.3±0.2 | 83±16 |
| E110 | 42±2 | 2.1±0.2 | 51±5 |
| T113 | 34±2 | 1.5±0.1 | 35±4 |
| G115 | 44±2 | 2.4±0.2 | 58±5 |
| Y119 | 45±2 | 2.4±0.1 | 60±5 |
| R121 | 50±2 | 2.9±0.2 | 74±6 |

**Table S3** Exchange parameters derived from global fit of ^15^N relaxation dispersion curves obtained for [80% ^2^H, 99% ^15^N-labeled] *apo* human I-BABP in 20 mM K-phosphate, 50 mM KCl, 0.05% NaN_3_, 10% D_2_O, pH=6.8 at 283 K.

| Residue | \|Δω\| (Hz) | R_ex_ (Hz) | Φ (Hz^2^) |
| --- | --- | --- | --- |
|  | **k_ex_ = 1467 ± 161 s^-1^ p_b_ = 4.4 ± 0.3 %** | | |
| G4 | 45±2 | 2.4±0.2 | 95±9 |
| E7 | 51±3 | 3.0±0.1 | 119±13 |
| S10 | 51±2 | 3.0±0.1 | 118±10 |
| E11 | 62±4 | 4.3±0.2 | 175±20 |
| E16 | 50±3 | 2.9±0.2 | 115±13 |
| S25 | 58±6 | 3.9±0.2 | 156±25 |
| K35 | 47±7 | 2.5±0.3 | 100±22 |
| I36 | 59±8 | 4.0±0.3 | 160±33 |
| Q45 | 64±4 | 4.7±0.1 | 192±21 |
| F47 | 47±5 | 2.6±0.2 | 104±17 |
| H52 | 50±4 | 2.9±0.2 | 116±15 |
| H57 | 48±6 | 2.7±0.1 | 107±20 |
| T60 | 46±5 | 2.5±0.1 | 99±17 |
| N61 | 45±7 | 2.4±0.3 | 95±22 |
| F63 | 43±5 | 2.2±0.2 | 86±15 |
| T73 | 102±9 | 10.9±0.4 | 483±69 |
| G88 | 71±8 | 5.6±0.3 | 231±40 |
| V91 | 87±6 | 8.3±0.3 | 353±42 |
| N96 | 85±8 | 8.0±0.4 | 337±50 |
| Y97 | 96±10 | 9.8±0.3 | 424±69 |
| H98 | 71±8 | 5.7±0.2 | 233±40 |
| Q99 | 68±8 | 5.1±0.2 | 211±38 |
| T100 | 81±10 | 7.3±0.2 | 306±57 |
| S101 | 66±9 | 4.9±0.3 | 202±41 |
| E102 | 49±7 | 2.8±0.2 | 111±24 |
| I103 | 47±7 | 2.5±0.2 | 100±22 |
| T118 | 56±6 | 3.6±0.2 | 144±24 |
| Y119 | 48±6 | 2.7±0.3 | 107±20 |

**Table S4** Exchange parameters derived from global fit of ^15^N relaxation dispersion curves obtained for [80% ^2^H, 99% ^15^N-labeled] *apo* human I-BABP in 20 mM K-phosphate, 50 mM KCl, 0.05% NaN_3_, 10% D_2_O, pH=8.0 at 283 K.

| Residue | \|Δω\| (Hz) | R_ex_ (Hz) | Φ (Hz^2^) |
| --- | --- | --- | --- |
|  | **k_ex_ = 1453 ± 194 s^-1^ p_b_ = 5.7 ± 0.4 %** | | |
| Y14 | 42±7 | 2.5±0.3 | 96±24 |
| E16 | 37±5 | 1.9±0.3 | 73±15 |
| K30 | 49±8 | 3.2±0.3 | 128±31 |
| F63 | 30±6 | 1.3±0.2 | 49±14 |
| I71 | 36±7 | 1.8±0.2 | 69±20 |
| T73 | 111±12 | 14.9±0.2 | 665±112 |
| A81 | 53±4 | 3.8±0.2 | 152±19 |
| T82 | 66±5 | 5.8±0.3 | 236±30 |
| L90 | 41±5 | 2.3±0.2 | 90±17 |
| V91 | 129±15 | 19.3±0.2 | 906±162 |
| N93 | 67±5 | 6.0±0.2 | 244±31 |
| Y97 | 52±7 | 3.6±0.3 | 144±29 |
| H98 | 82±9 | 8.8±0.2 | 367±63 |
| Q99 | 55±5 | 4.1±0.3 | 164±24 |
| T100 | 75±7 | 7.3±0.2 | 300±45 |
| S101 | 46±6 | 3.0±0.2 | 117±23 |
| T113 | 56±7 | 4.3±0.2 | 169±32 |
| Y119 | 68±7 | 6.2±0.3 | 251±41 |
